# Quantification of dissolved H_2_ and continuous monitoring of hydrogen-rich water for haemodialysis applications: An experimental study

**DOI:** 10.1177/03913988211070588

**Published:** 2022-01-25

**Authors:** Foivos Leonidas Mouzakis, Lal Babu Khadka, Miguel Pereira da Silva, Khosrow Mottaghy

**Affiliations:** 1 Institute of Physiology, RWTH Aachen University Hospital, Aachen, Germany; 2 Laboratory of Membrane Processes CeFEMA, Instituto Superior Técnico, Universidade de Lisboa, Lisbon, Portugal

**Keywords:** Haemodialysis, artificial kidney, apheresis & detoxification techniques, sensors, hydrogen, extracorporeal circulation, hydrogen water monitoring system

## Abstract

The prevalence of oxidative and inflammatory stress in end-stage renal disease (ESRD) patients has often been associated with chronic haemodialysis therapies. Over the past decades, several reports have shown the potential of hydrogen molecule as an antioxidant in the treatment of various medical conditions in animal models, as well as in pilot studies with human patients. Recently, a hydrogen-enriched dialysate solution has been introduced, holding promise in reducing the oxidative and/or inflammatory complications arising during haemodialysis. To this end, a standardised measuring method to determine the levels of hydrogen in dialysate and subsequently in blood is required. This study explores the possibility of quantifying hydrogen concentration using a novel contactless sensor that detects dissolved hydrogen in liquids. An experimental circuit is assembled to validate the sensitivity and accuracy of the hydrogen monitoring system (Pureron Japan Co., Ltd) through in vitro investigations with physiological solutions. Measurements of dissolved molecular hydrogen concentration are corroborated by an established oxygen sensor providing continuous partial pressure readings. The relationship between the applied H_2_ content in the gaseous mixture and the H_2_ concentration value at equilibrium is linear. At the same time, the hydrogen monitoring system has a rather long response time, and its readings seem to slightly diverge from sensor to sensor as well as at different temperatures. For this reason, a sensor recalibration might be necessary, which could become part of the product’s ongoing development. Nevertheless, the aforementioned minor deficiencies can be mostly considered negligible in applications such as haemodialysis.

## Introduction

Haemodialysis (HD) is a stationary clinical treatment used in patients in the late stages of Chronic Kidney Disease (CKD) consisting of up to three regular sessions lasting 4–5 h each,^1^ during which, blood flowing through an extracorporeal circulation (ECC) system is continuously purified by means of a capillary membrane hemodialyzer.^2,3^ The production of reactive oxidative species (ROS) that are responsible for a state of oxidative stress is amongst the various pathogenic mechanisms associated with renal disease.^4^ Oxidative stress is present from the very early stages of CKD and, as the production of ROS gradually intensifies with renal impairment, it gets further aggravated by HD procedures.^5^ At the same time, the frequency and duration of the HD therapy combined with the exposure of blood to the ECC circuit’s foreign surfaces and pumping devices are known to induce systemic inflammation.^6,7^ This inflammatory response triggers oxidative stress and reduces cellular antioxidant capacity, resulting in an overproduction of free radicals that permanently hinder the function of cell membrane fatty acids and proteins.^8^ The presence of excess free radicals may lead to DNA damage and mutation thus constituting a predisposing factor for cancer and age-related disorders.^9^ In consequence, the combination of enhanced oxidative stress and inflammation poses a real threat with severe repercussions associated with the occurrence of cardiovascular events and death amongst chronic HD patients.^10,11^

In that regard, the use of antioxidant and anti-inflammatory agents such as molecular hydrogen (H_2_) has proven to be beneficial against oxidative stress-mediated disorders and inflammatory diseases, on account of its vital role in certain biological molecular mechanisms.^12^ These mechanisms include selective scavenging of strong oxidants (e.g. hydroxyl radicals), and regulation of signal transduction and gene expression.^8,13^ Hydrogen is highly diffusible and easily reaches membrane-bound cell organelles, such as mitochondria and nuclei – considered the primary sites of ROS generation and DNA damage respectively^13^ – and it can even traverse the blood brain barrier, which is impermeable to most antioxidant compounds.^14^ Dole et al.^15^ in 1975, has demonstrated that H_2_ possesses therapeutic properties contributing to the elimination of cytotoxic radicals. Since then, it has been successfully used as an antioxidant in the treatment of cerebral and cardiac ischaemia-induced injuries,^16–19^ pulmonary oedema due to extensive burns,^20^ and during organ transplantation^21^ in several animal models. Spulber et al.^22^ further demonstrated that hydrogen stimulates anti-inflammatory gene expression in mice, thus improving their recovery rate. Similar studies on animals support the beneficial usage of hydrogen against chronic inflammation in the case of hepatitis, colitis, pancreatitis and sepsis.^23–26^ So far several modes of H_2_ delivery have been attempted, such as inhalation,^15,16,18,19,21^ ingestion of hydrogen-rich water^8,22,27^ and injection with hydrogen-dissolved saline.^17,20,28^ Recently, a novel HD system delivering an H_2_-enriched dialysis solution has been implemented and reported a significant suppression of oxidative stress markers, including a reduction of high blood pressure levels in patients undergoing the treatment.^1,29–31^ Similarly, oral administration of H_2_-enriched water has effectively limited the oxidative stress response in a pilot study with rheumatoid arthritis patients,^27^ and has improved lipid and glucose metabolism in patients with type 2 diabetes mellitus or impaired glucose tolerance.^32^

Although the use of H_2_ is generally recognised to have beneficial anti-oxidative and anti-inflammatory biological effects, little attention has been given to experimental approaches that can accurately trace its clinical dosage in vivo, namely in patients’ bloodstream, which is the main focus of the present work. For this purpose, a novel contactless H_2_ sensor capable of real-time monitoring of hydrogen concentration in liquids is coupled to an in vitro ECC setup to characterise and validate its performance in the quantification of dissolved H_2_ in circulating blood substitute solutions.

## Materials and experimental methods

**Figure 1.**
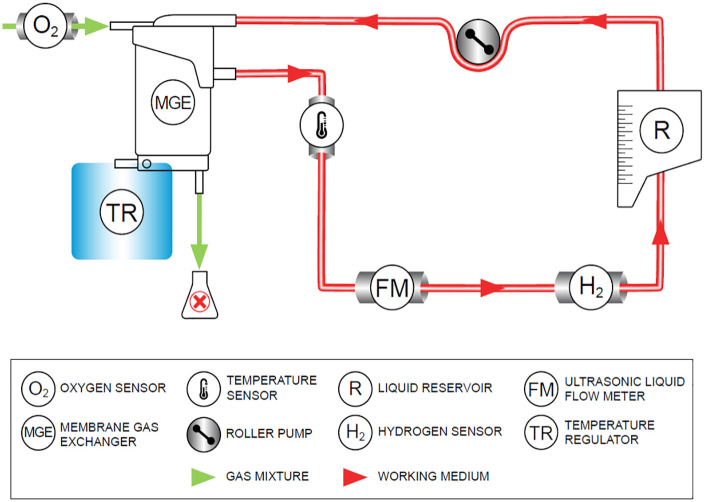
Graphical representation of the experimental setup.

The continuous measurement of dissolved H_2_ concentration in circulating working media has been conducted in a laboratory closed-loop circuit equipped with a novel in-line contactless hydrogen monitoring device. Figure 1 shows a scheme of the assembled system used in the quantification of dissolved molecular hydrogen in physiological solutions.

A peristaltic pump generates a constant feed flow rate of $0.5\;l\;min^{-1}$ , monitored by an ultrasonic flowmeter, throughout all the experimental runs. In this work, saline solution with a NaCl concentration of $9\;g\;l^{-1}$ , and water were used as circulating working media. Following the pump, the fluid is propelled through a membrane gas-exchanger device (MGE) where it is continuously enriched in molecular hydrogen by feeding H_2_/Air gaseous mixtures into the MGE’s gas-side (Hydrogen 5.0, Linde AG, Germany). Other modes of gas delivery into the working medium were explored, such as direct sparging of gas mixtures into the feed reservoir, but revealed gas leakage from the system. To overcome this issue, a membrane blood oxygenator (Medos Hilite 800 LT, Xenios AG, Germany) acting solely as an MGE, was employed to promote an efficient mass transfer, while assuring minimum leakage of gaseous H_2_ from the working medium. Furthermore, the MGE’s gas-side exhaust is placed under a laminar flow hood to successfully dispose of gaseous H_2_. The MGE device is connected to a temperature regulator (heat exchanger) that maintains a constant temperature of 20℃. Finally, the enriched liquid flows back to the feed reservoir. The concentration of dissolved hydrogen, $\left[H_{2}\right]$ , with an uncertainty of ±0.5ppb , is continuously quantified by a contactless sensor connected to HWMS-Mark III, by Pureron Japan Co., Ltd, a hydrogen water monitoring system calibrated at 20℃. The sensor is clamped onto the circuit tubing downstream of the MGE, and permits the quantification of H_2_ concentration in the circulating solution, in the $0\leq \left[H_{2}\right]\leq 1.2\;ppm$ range. Conjointly, the partial pressure of oxygen, $p_{O_{2}}$ , in the fed gaseous mixture is monitored continuously by an in-line pressure sensor (FDO2, Pyroscience GmbH, Germany) placed at the gas inlet.

The extensive research conducted so far on the remedial properties of molecular hydrogen, has been consulted in advance of carrying out any experiment with the above described setup. Fang et al.^20^ used 0.6mmol/L hydrogen-rich saline for infusions, whereas Nakayama et al.^1,29–31^ has reported concentrations of dissolved hydrogen in dialysate ranging from 30 to 210ppb in HD therapies. Therefore, concentrations of dissolved molecular hydrogen in the range of $20\leq \left[H_{2}\right]\leq 250\;ppb$ are employed for the purposes of this study.

Molecular hydrogen does not react with most compounds (including oxygen gas) exhibiting an inert gas behaviour at room/body temperature, if no catalysts are present.^33^ Therefore, mixing hydrogen with air to facilitate its indirect traceability through the partial pressure of the other gaseous components, should not raise any safety questions. Table 1 shows relevant physicochemical data regarding the gaseous mixture components present in this study.

**Table 1.** Physical and chemical properties of the components in the H_2_/Air gaseous mixture.

	H_2_	O_2_	CO_2_	N_2_
Average molecular mass [g mol^−1^]	2.016	31.998	44.009	28.0134
Density (STP) [g l^−1^]	0.08988	1.429	1.977	1.2506
Boiling point [K (°C)]	20.271 (−252.879)	90.188 (−182.962)	194.686 (−78.464)	77.355 (−195.795)
Diffusion coefficient in air (273.15 K, 1 atm) [cm^2^ s^−1^]^34^	1.604	0.192	0.106	0.155
Diffusion coefficient in H_2_O (298.15 K, 1 atm) [cm^2^ s^−1^]^35^	4.50 × 10^−5^	2.10 × 10^−5^	1.92 × 10^−5^	1.88 × 10^−5^
Henry’s Law constant (298.15 K) [mol m^−3^ Pa^−1^]^36^	7.8 × 10^−6^	1.3 × 10^−5^	3.3 × 10^−4^	6.4 × 10^−6^

Under the assumption of an H_2_/Air ideal gas mixture, the H_2_ content can be indirectly monitored by the sensor through the measurement of the partial pressure of oxygen following Dalton’s law of partial pressure (1, 2)^37^:

(1) $$p_{i}+p_{i+1}+\ldots +p_{n}=P_{Total}$$

(2) $$p_{i}=y_{i}\times P_{Total}$$

Where $p_{i}$ and $y_{i}$ correspond to the partial pressure and the volume fraction of component i in the gaseous mixture respectively; and $P_{Total}$ is the total pressure. This way, it is to be expected that as the content of H_2_, $y_{H_{2}}$ , increases, so does its partial pressure, $p_{H_{2}}$ , to the detriment of the partial pressure of the remaining components in the gaseous mixture, including that of oxygen, $p_{O_{2}}$ .

All the data obtained was downsampled to experimental points acquired every 3 s throughout each experiment.

The data on $p_{O_{2}}$ is corrected to 20℃ using the solubility of oxygen in pure water, $s_{O_{s}\to H_{2}O}$ , by a relationship of the type (3):

(3) $$p_{O_{2}}\;at\;20^{{}^{\circ}}C\;\;\;\;\;\;\;=\frac{s_{O_{s}\to H_{2}O}\;at\;given\;T^{{}^{\circ}}C\;\;\;\;\;\;}{s_{O_{s}\to H_{2}O}\;at\;20^{{}^{\circ}}C\;\;\;\;\;\;}\times \;p_{O_{2}}at\;T^{{}^{\circ}}C\;\;\;\;\;\;$$

## Results

Two successive experimental runs ( n=1,2 ) are performed in the previously described setup to define the operating conditions. In the first experimental run ( n=1 ), the hydrogen flow rate is equal to $Q_{H_{2}}=53\pm 2\;ml\;\min^{-1}$ and a steady-state H_2_ concentration is reached at H_2_:Air gas flow ratios of 1:40, 1:20 and 1:10, which correspond to a vol% of H_2_ in the feed gas mixture equal to 2.67±0.07% , 5.4±0.15% and 10.4±0.5% , respectively. Likewise, in the second experimental run ( n=2 ), the hydrogen flow rate is $Q_{H_{2}}=53\pm 1\;ml\;\min^{-1}$ and the steady-state is reached at H_2_:Air gas flow ratios of 1:40, 1:20 and 1:10, representing a vol% of H_2_ in the feed gas mixture of 2.7±0.05% , 5.3±0.09% and 10.5±0.3% respectively.

The oxygen sensor registers a partial pressure of oxygen in the feed gas mixture of 145.52 , 141.28 and 134.84mmHg respectively for the aforementioned H_2_:Air ratios of 1:40, 1:20 and 1:10. The sensor’s reading when the gas mixture consists only of air amounts to 149.47mmHg .

The theoretical pO_2_ values for the given H_2_:Air ratios (1:40, 1:20 and 1:10) are 145.48 , 141.54 and 133.77mmHg respectively. Hence, the experimental data display good agreement with the predicted values across the measurement range, validating the oxygen sensor’s accuracy.

**Figure 2.**
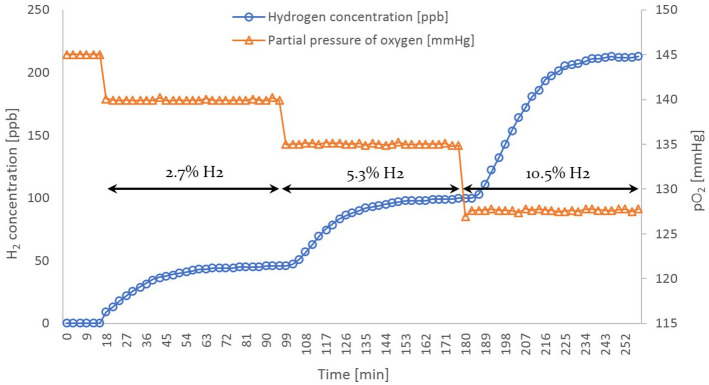
Evolution of dissolved hydrogen concentration and pO_2_ for the different operating conditions.

The HWMS reports the following values of molecular hydrogen concentration once steady state has been reached for each of the established feed gas hydrogen contents: 47±2% , 100±3% and 211±3% . Figure 2 depicts the course of pO_2_ and H_2_ concentration over time under the given experimental conditions.

Throughout the experimental run, regardless of the operating condition, the HWMS demonstrates a hysteresis of roughly 50 min until the equivalent equilibrium value is achieved. The oxygen sensor in contrast, exhibits high responsiveness, rapidly registering changes in partial pressure, thus corroborating its function as a reference measurement method. To put this disparity in response time into perspective, the HWMS lags by more than three orders of magnitude behind the oxygen sensor.

**Figure 3.**
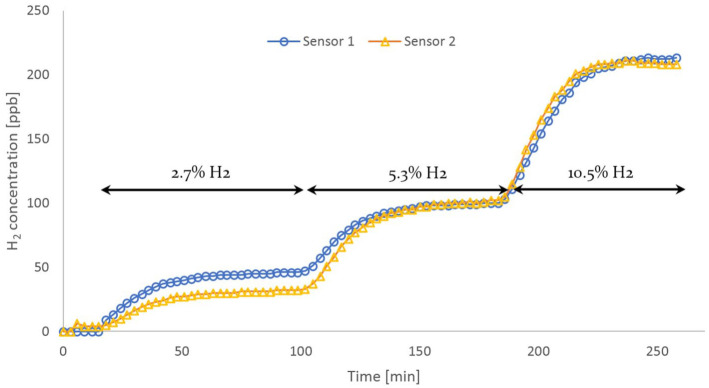
Hydrogen concentration profiles of two HWMS devices under the same operating conditions.

To confirm the above results, a second HWMS unit has been introduced in the experimental circuit, with its sensor clamped right next to the pre-existing one. A second round of investigations have been conducted, based on the established protocol. As Figure 3 reveals, the measurement profiles of both HWMS devices follow comparable courses throughout the experimental procedure, under identical operating conditions. The values at equilibrium do not deviate significantly in either profile. At higher concentrations, namely at t=180min and t=250min , the divergence is equal to 1% (for H_2_ content of 5.3% ) and 1.4% (for 10.5 vol% H_2_) respectively. However, a 30% disparity between both profiles can be observed for 60<t<100min min, during the initial operating condition ( 2.7 vol% H_2_). Interestingly, the sensors’ response time becomes shorter at higher H_2_ concentrations, presumably indicating a relationship between sensitivity and responsiveness.

Figure 3 underlines the reproducibility of the test protocol and accentuates the hydrogen sensors’ sensitivity and precision.

The concept behind the development of HWMS and the application of H_2_-rich dialysate in HD therapy involves different liquids at diverse temperatures. Hence, emphasis ought to be placed on investigating hydrogen’s diffusivity and solubility at different temperatures. To this end, a third HWMS unit, factory-calibrated at 36℃, is introduced into the circuit, placed alongside the original one (factory-calibrated at 20℃) in the test circuit, with the intent of comparing their readings under identical conditions. On this occasion, only two operating conditions shall be tested, with feed gas hydrogen contents (H_2_ vol%) of 3.5% and 6.6% , at two temperature levels (20℃ and 36℃).

**Figure 4.**
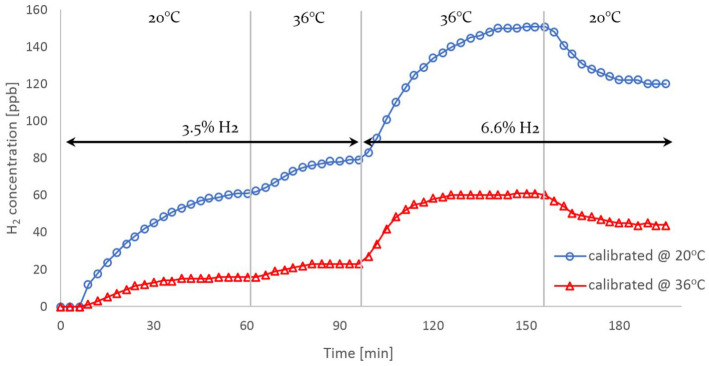
Hydrogen concentration at different temperatures as recorded by two HWMS devices calibrated at 20℃ and 36℃ respectively.

As Figure 4 illustrates, the steady state values registered by each sensor at its operating temperature do not coincide. In fact, the initial discrepancy between the two sensors’ measurements further distends with rising temperature and for higher hydrogen content in the feed gas. Moreover, the dissolved hydrogen concentration measurements fluctuate when temperature changes, although hydrogen content in the feed gas remains constant. This behaviour suggests once again that the sensors’ sensitivity might be affected by factors such as temperature or higher H_2_ concentrations. Even so, this phenomenon is puzzling, since it directly contradicts the principle of decreasing solubility with rising temperature, and therefore requires further investigation.

**Figure 5.**
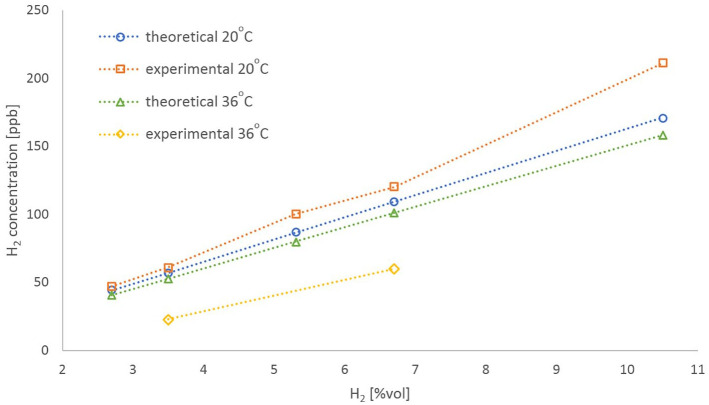
Experimental versus theoretical data of dissolved hydrogen concentration for all the operating conditions.

To address the sensitivity and accuracy issues of the HWMS units, a theoretical approach has been pursued that would determine the actual concentration of dissolved hydrogen in the working medium for the given operating conditions. Figure 5 represents the experimental data obtained from the various investigations carried out, accompanied by the theoretical values of dissolved hydrogen concentration, for all the operating conditions implemented in this study. As one may appreciate, the relative error between measured and estimated values increases with rising temperatures/concentrations, as discussed earlier. A factory recalibration of the sensors might moderate this trend and reduce the overall measurement error.

## Discussion

This study has been realised about HWMS, a novel contactless H_2_ sensor for real-time measurements of dissolved molecular hydrogen concentration in liquids. An experimental setup based on extracorporeal circulation principles has been assembled for optimal delivery of H_2_/Air gaseous mixtures in the working medium. The results demonstrate the measuring capabilities of HWMS, as well as some shortcomings, chief among which is the sluggish response time. Although high responsiveness is a desired trait among sensors of all kinds, its scantiness does not necessarily constitute an obstacle for HD applications, since the treatment itself is a lengthy procedure, and the hysteresis of the sensor becomes negligible in this context. Accuracy, on the other hand, is a crucial parameter for any measuring apparatus, hence the fluctuating readings observed at higher H_2_ concentrations ought to be addressed forthwith. Similar inadequacies in accuracy have been noted at higher temperatures at even greater rates, even from sensors calibrated at these conditions. The majority of the above mentioned plights could possibly be mitigated to some extent by defining new/case-specific calibration curves for all the HWMS devices. This ought to suppress the divergence in H_2_ concentration measurements between sensors, and may also diminish the relative error at higher temperatures.

Further research, and implementation of additional direct/indirect methods of measuring hydrogen concentration in gas and/or liquid phase (e.g. gas chromatography),^18,38,39^ would provide evidence of the HWMS system’s accuracy and reliability. In addition, in vitro investigations with animal blood (e.g. porcine, due to its physiological similitude to human blood) could assist in investigating hydrogen’s palliative properties in extracorporeal settings. This may prove particularly enlightening since hydrogen’s anti-oxidative and anti-inflammatory action might be appealing to a wide range of disciplines, especially when one takes into account blood trauma and the pro-inflammatory influence of commonly used medical devices such as pumps, filters and catheters, where cell distribution induced by diversified flow patterns is prevalent.^40–42^
